# Genome-Wide Analysis of GLD-1–Mediated mRNA Regulation Suggests a Role in mRNA Storage

**DOI:** 10.1371/journal.pgen.1002742

**Published:** 2012-05-31

**Authors:** Claudia Scheckel, Dimos Gaidatzis, Jane E. Wright, Rafal Ciosk

**Affiliations:** Friedrich Miescher Institute for Biomedical Research, Basel, Switzerland; Harvard University, United States of America

## Abstract

Translational repression is often accompanied by mRNA degradation. In contrast, many mRNAs in germ cells and neurons are “stored" in the cytoplasm in a repressed but stable form. Unlike repression, the stabilization of these mRNAs is surprisingly little understood. A key player in *Caenorhabditis elegans* germ cell development is the STAR domain protein GLD-1. By genome-wide analysis of mRNA regulation in the germ line, we observed that GLD-1 has a widespread role in repressing translation but, importantly, also in stabilizing a sub-population of its mRNA targets. Additionally, these mRNAs appear to be stabilized by the DDX6-like RNA helicase CGH-1, which is a conserved component of germ granules and processing bodies. Because many GLD-1 and CGH-1 stabilized mRNAs encode factors important for the oocyte-to-embryo transition (OET), our findings suggest that the regulation by GLD-1 and CGH-1 serves two purposes. Firstly, GLD-1–dependent repression prevents precocious translation of OET–promoting mRNAs. Secondly, GLD-1– and CGH-1–dependent stabilization ensures that these mRNAs are sufficiently abundant for robust translation when activated during OET. In the absence of this protective mechanism, the accumulation of OET–promoting mRNAs, and consequently the oocyte-to-embryo transition, might be compromised.

## Author Summary

One of the most striking developmental events is the oocyte-to-embryo transition that, in the absence of Pol II–dependent transcription, depends on regulated translation of maternal mRNAs. Prior to their activation, these maternal mRNAs need to be “stored" in the egg cytoplasm in a repressed but stable form. Surprisingly little is known about how the stored mRNAs are stabilized. The STAR family of RNA–binding proteins includes the *C. elegans* GLD-1, which controls many aspects of germ cell development. To obtain a comprehensive picture of GLD-1–dependent mRNA regulation, we performed a genome-wide survey of translational repression and mRNA stability of GLD-1 targets. This uncovered a potential role of GLD-1 in mRNA storage, as we found that GLD-1 both represses and stabilizes a subpopulation of its targets. The stabilization also involves a DDX6-like RNA helicase, CGH-1, which is a component of repressive germ granules and processing bodies. Remarkably, the GLD-1 and CGH-1 stabilized mRNAs encode regulators of the oocyte-to-embryo transition, providing an insight into how these functionally related mRNAs are specifically stabilized during germ cell formation. These findings have potential implications for oocyte quality and reproductive fitness, and for mRNA storage in other cell types such as neurons.

## Introduction

The oocyte-to-embryo transition (OET), which encompasses oocyte maturation, ovulation, fertilization, and early embryogenesis, occurs while Pol II dependent transcription is globally repressed. This is why OET is largely driven by maternal mRNAs that are stored in the egg cytoplasm in a repressed but, importantly, also stable form [1]. In contrast to translational repression, stabilization of repressed mRNAs remains little understood. In *Xenopus* oocytes, mRNA stability is attributed to a global inhibition of decapping activity [2], [3]. On the other hand, in developing *Drosophila* oocytes, stabilization of the *bicoid* mRNA depends on the binding of a specific protein, BSF [4]. This suggests that, at least in some species, global inhibition of mRNA decay is not a general feature of oogenesis and thus mechanisms stabilizing specific germline messages might exist.

In *C. elegans*, the DDX6-like RNA helicase, CGH-1, associates with a large number of germline mRNAs [5]. Some of these mRNAs are less abundant in CGH-1 (-) germ cells, suggesting that this helicase plays a role in mRNA stabilization [5]. The DDX6-like helicases are present in various cytoplasmic ribonucleoprotein (RNP) particles such as processing (P) bodies [5]–[14]. In the *C. elegans* germ line, CGH-1 localizes to P granules, which are associated with the nuclear envelope, and to P body-like cytoplasmic granules [15]. In contrast to P bodies, the latter granules seem to be largely devoid of RNA decay enzymes and have thus been proposed to serve as vehicles of mRNA storage, which is consistent with a role of CGH-1 in mRNA stabilization, [5], [16]–[22]. However, because somatic P body formation is thought to be the consequence, not the cause, of mRNA repression [23], [24], the functional significance of these RNA granules for mRNA stabilization remains to be demonstrated.

**Figure 1:**
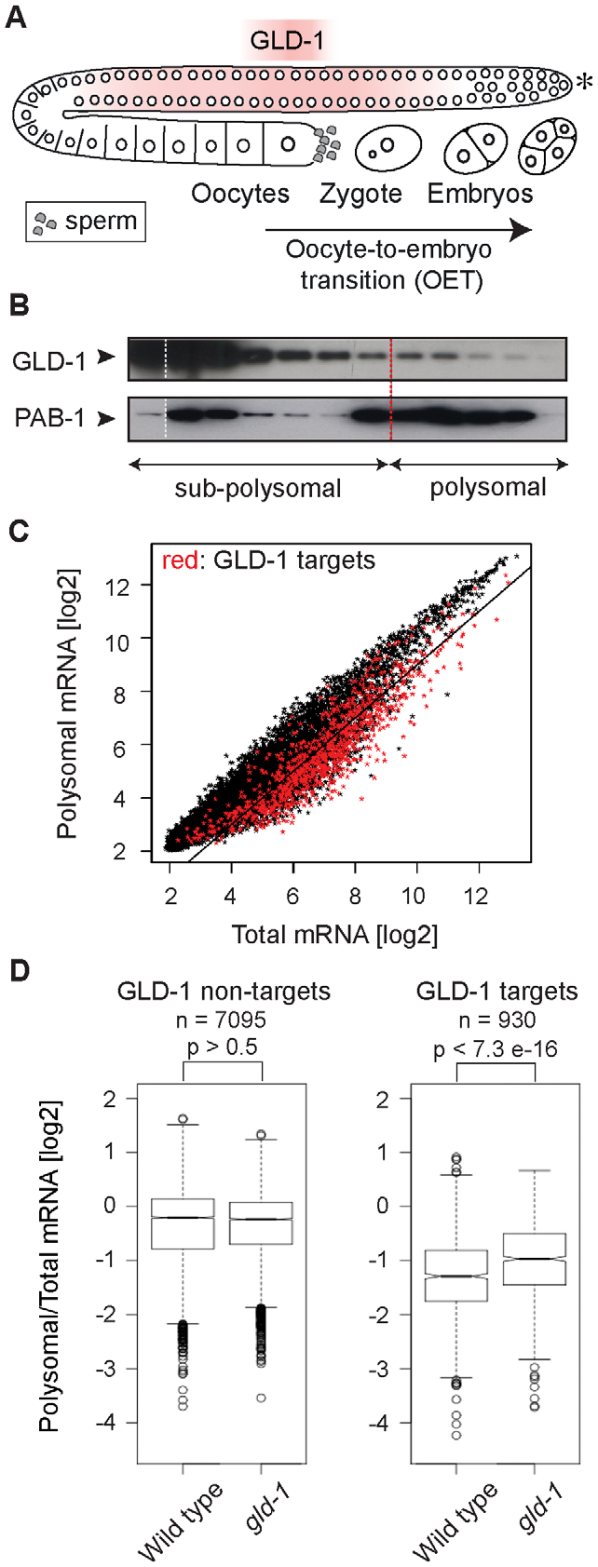
GLD-1 has a widespread role in repressing translation. (A) Schematic of a *C. elegans* gonad and the oocyte-to-embryo transition (OET). The distal-most gonad contains stem cells and is marked here and in subsequent figures by an asterisk. The medial gonad contains germ cells undergoing meiosis that are expressing GLD-1. Growing oocytes are present in the proximal gonad. OET, including ovulation, fertilization, and early stages of embryonic development, occurs while Pol II transcription is globally repressed. (B) GLD-1 mostly co-fractionates with non-translated mRNAs. Distribution of GLD-1 and PAB-1 proteins, detected with specific antibodies by western blotting, between fractions from a polysome profiling experiment. (C) Identification of non-translated mRNAs, including many GLD-1 targets, by a transcriptome-wide survey of translational repression. Polysomal and total mRNAs from wild-type animals were analyzed by polysome profiling followed by microarray-based detection. Polysomal mRNA levels were plotted against total mRNA levels. Each dot in this and subsequent scatter plots represents a single mRNA. Transcripts that are more than two-fold depleted from polysomal fractions are below the black line. GLD-1 targets are colored in red (see Figure S1D). (D) GLD-1 represses translation initiation. Box plots represent the distribution of polysomal/total mRNA ratios for GLD-1 targets and non-targets in wild type and *gld-1(q485)* mutants (called simply *gld-1*). The polysomal/total mRNA ratio of non-targets is similar in wild-type and *gld-1* animals (left panel). In contrast, GLD-1 targets shift to polysomes in *gld-1* mutants (right panel). Sample sizes (n) and p values are indicated. The p values were calculated with a t test.

Here, we report the *C. elegans* STAR-protein GLD-1 as a potential player in maternal mRNA storage. GLD-1 is expressed in the medial gonad (Figure 1A), where it promotes meiosis, oogenesis, and maintenance of germ cell identity by repressing the translation of diverse mRNAs [25]–[30]. Recently, we have shown that GLD-1 associates with hundreds of germline transcripts, and that this association is determined by the number and strength of 7-mer GLD-1 binding motifs (GBMs) within untranslated regions (UTRs) [31]. To understand how GLD-1 regulates its mRNA targets, we undertook a functional genomics approach. By transcriptome-wide polysome profiling, we found that GLD-1 has a widespread role in repressing translation. Our results also suggest that GLD-1 stabilizes many targets, which additionally involves the DDX6-like RNA helicase CGH-1. Because the stabilized mRNAs encode proteins critical for OET, and their stability appears to be important for efficient accumulation in oocytes, GLD-1 dependent mRNA storage might be important for a successful oocyte-to-embryo transition.

## Results

### GLD-1 has a widespread role in repressing translation

It is currently unknown how GLD-1 represses translation. By ‘polysome profiling’, in which poly-ribosomes (polysomes) are separated from single ribosomes and ribosomal subunits by sucrose density gradient ultracentrifugation, two of the GLD-1 targets, *tra-2* and *pal-1*, have been suggested to be repressed at the initiation or elongation stage of translation, respectively [30], [32]. To globally examine the effect of GLD-1 on the translation of its targets, we performed polysome profiling on a transcriptome-wide scale. In general, while polysomal fractions contain translated mRNAs (as well as mRNAs repressed at the elongation or termination stage of translation), sub-polysomal fractions contain poorly translated mRNAs and transcripts repressed at the initiation stage of translation (Figure S1A–S1C). To determine the distribution of GLD-1 between fractions, we used monoclonal antibodies raised against GLD-1, and, as a control, against the translational activator polyA-binding protein, PAB-1. As expected, PAB-1 was enriched in polysomal fractions (Figure 1B). In contrast, the majority of GLD-1 was present in the sub-polysomal fractions (Figure 1B). By comparing polysomal and total mRNA levels by microarray analysis, we found that also most GLD-1 targets (Table S1 and Figure 1C and Figure S1D; mRNAs more than 3-fold enriched in GLD-1 IPs; also [31]) were enriched in sub-polysomal fractions (Figure 1C; GLD-1 targets are in red; transcripts more than 2-fold depleted from polysomes, including 64% of GLD-1 targets, are below the black line). To examine GLD-1 dependent repression, we tested whether GLD-1 targets shift to polysomal fractions in *gld-1(q485)* null mutant worms (hereafter called *gld-1* mutants). Because *gld-1* mutants develop germline tumors, we only examined young adults, in which the gonads contained large numbers of pachytene cells and only few ectopically proliferating cells [26], [33]. To collect sufficient quantities of mutant animals, *gld-1* homozygous mutants were separated from heterozygous animals, carrying a GFP-tagged balancer, by fluorescence-activated sorting. Expectedly, we found that the loss of GLD-1 had little effect on the polysomal/total mRNA ratio of non-GLD-1 targets and of targets of an unrelated RBP, FBF [34] (Figure 1D and Figure S1E). In contrast, the loss of GLD-1 caused GLD-1 targets to shift to polysomes (Figure 1D; p<7.3e−16; p values were calculated with a t test). While the polysomal/total mRNA ratio of GLD-1 targets remained relatively low in *gld-1* mutants compared to non-targets (possibly due to residual repression by other RBPs as has been observed for several GLD-1 targets [30], [35], [36]), these results collectively suggest that, although additional mechanisms may exist and contribute to repression, GLD-1 binding inhibits translational initiation.

### GLD-1 is required for the accumulation of many mRNA targets

**Figure 2:**
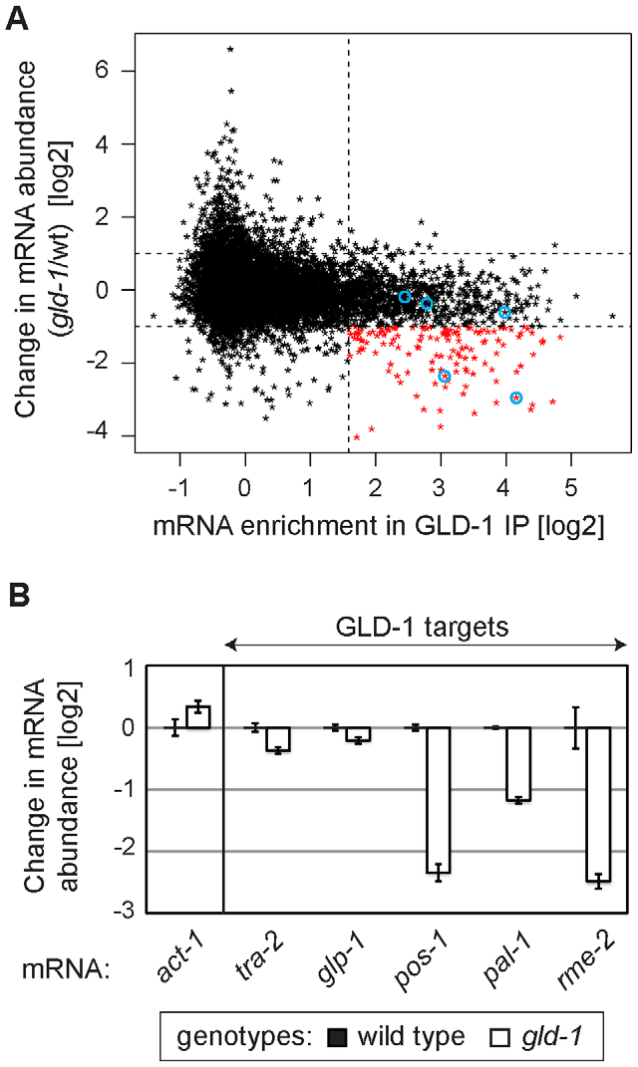
GLD-1 is required for the accumulation of many mRNA targets. (A) Many GLD-1 targets are less abundant in *gld-1* mutants. Abundance of mRNAs in dissected wild-type and *gld-1* gonads was measured by microarrays. The change in mRNA abundance in *gld-1* mutants was plotted against GLD-1 IP enrichment. The vertical line separates non-targets on the left from GLD-1 targets on the right. Horizontal lines demarcate a 2-fold change of mRNA abundance in *gld-1* gonads. mRNAs verified in Figure 2B are encircled in blue. (B) A decrease in the levels of several GLD-1 targets was independently confirmed by RT-qPCR. Transcripts are arranged according to their GLD-1 IP enrichment in Figure 2A (the blue circles begin on the left with *tra-2* and end with *rme-2* on the right). The levels of indicated mRNAs were normalized to *tbb-2* mRNA. Shown are changes in mRNA levels in *gld-1* mutants relative to wild-type animals. Error bars here, and in subsequent figures, represent SEM of at least three biological replicates.

Several GLD-1 targets have been observed by others to be less abundant in *gld-1* mutants [26]–[29], [37], [38]. To globally determine a potential function of GLD-1 in mRNA stabilization, we analyzed the abundance of mRNAs in wild-type and *gld-1* mutant gonads by microarray analysis. We then compared changes in the mRNA abundance in *gld-1* mutants to GLD-1 binding (Table S1 and Figure 2A; the vertical dotted line separates non-targets on the left from presumed GLD-1 targets on the right and the horizontal lines demarcate a 2-fold change of mRNA abundance). We observed that a subset of GLD-1 targets (14%) were less abundant in *gld-1* mutants (Figure 2A, transcripts marked in red, those encircled in blue were confirmed by RT-qPCR in 2B). Because these mRNAs also tend to shift to polysomes in *gld-1* mutants (Figure S2), our results suggest that GLD-1 may control both their repression and stability.

### GLD-1 interacts with conserved components of germline granules and P bodies

**Figure 3:**
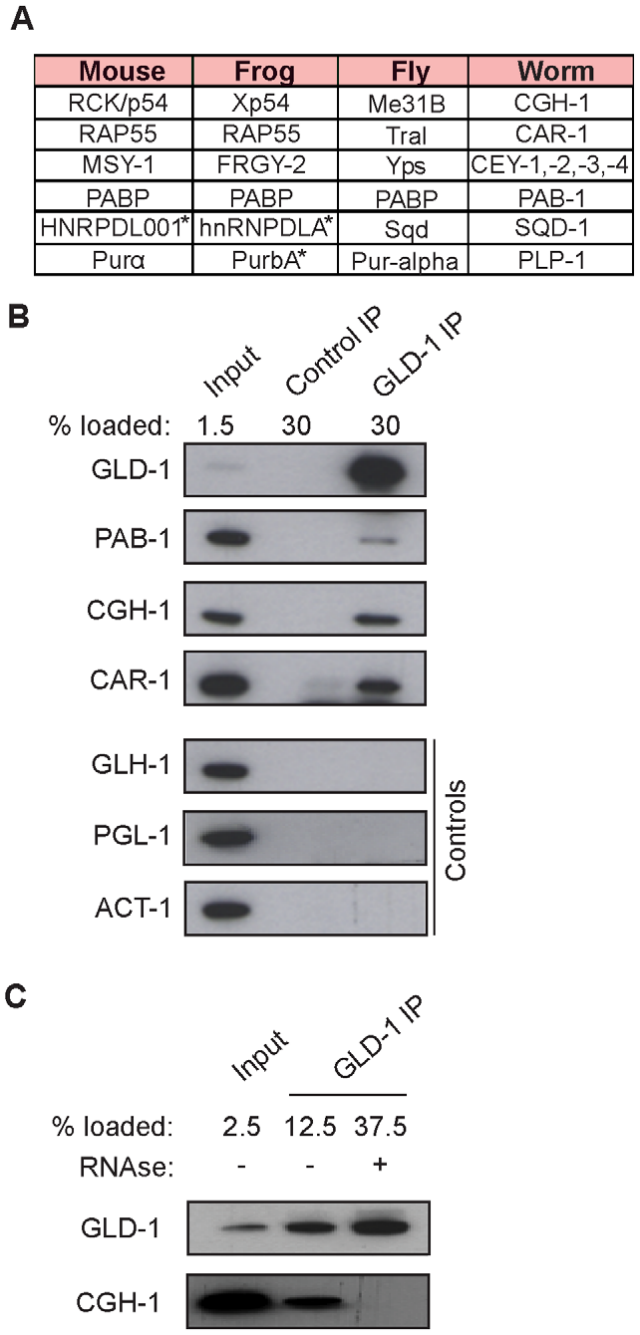
GLD-1 interacts with conserved components of RNA granules. (A) A table summarizing top GLD-1 interacting proteins and their counterparts in other species. Shown are GLD-1 co-precipitated proteins (identified by mass spectrometry) and their homologs. Proteins marked with asterisks are predicted based on available transcripts. (B) Confirmation of some interactions by western blot analysis of GLD-1 IPs. GLH-1/Vasa, PGL-1, and ACT-1/actin are negative controls. (C) The interaction between GLD-1 and CGH-1 depends on RNA. RNAse treatment of GLD-1 IPs prevents CGH-1 from co-immunoprecipitating with GLD-1.

To investigate potential partners of GLD-1 in mRNA stabilization, we immunopurified GLD-1 and analyzed co-purifed proteins by mass spectrometry. Top proteins most enriched in GLD-1 immunoprecipitates (IPs), together with their counterparts in other animals, are listed in Figure 3A (also see Figure S3A). These include the DDX6-like RNA helicase CGH-1, the Y-box proteins CEY-1-4, the Sm-like domain protein CAR-1, and the cytoplasmic polyA binding protein PAB-1, all of which are conserved components of repressive RNPs in germ cells and somatic cells, and which have been previously shown to interact with each other [5], [8]–[14], [22]. Using available antibodies, we confirmed by western blot analysis that the interactions between GLD-1 and CGH-1, CAR-1, and PAB-1, were specific (Figure 3B). CGH-1 has previously been implicated in the stabilization of at least some maternal mRNA [5], which is why we pursued its interaction with GLD-1 further. We observed that the GLD-1/CGH-1 interaction was dependent on RNA (Figure 3C) and confocal microscopy revealed that only a minor fraction of GLD-1 co-localized with CGH-1 in the germline cytoplasm (Figure S3B).

### GLD-1 and CGH-1 are required for the accumulation of common mRNAs

**Figure 4:**
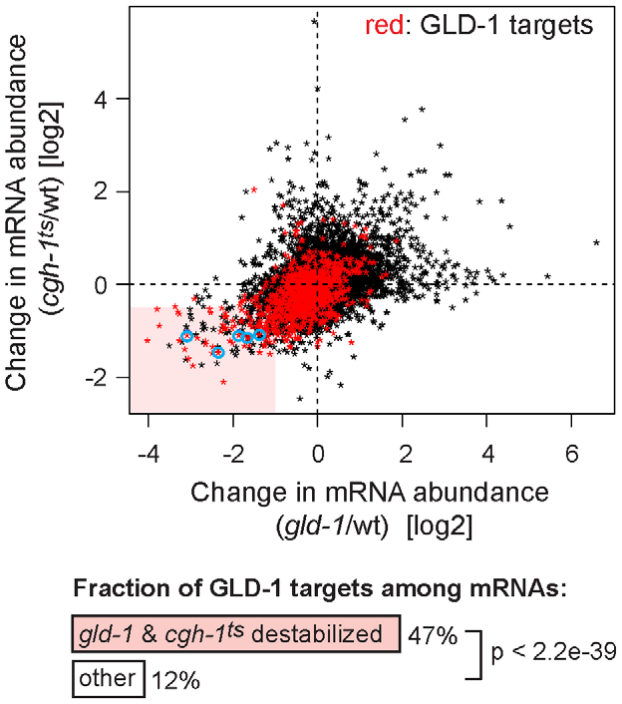
GLD-1 and CGH-1 are both required for the accumulation of a subset of GLD-1 targets. mRNA levels in dissected wild-type, *gld-1*, and *cgh-1^ts^* gonads were measured by microarrays. The change in mRNA abundance in *cgh-1^ts^* mutants was plotted against the change in mRNA abundance in *gld-1* mutants. GLD-1 targets are marked in red. mRNAs verified in Figure 6A and Figure S4A and S4C are encircled in blue. The light red rectangle contains mRNAs whose abundance drops the most in both mutants. These mRNAs are 4-fold enriched for GLD-1 targets compared to all germline mRNAs (p<2.2e−39, t test).

Despite the indirect (RNA-mediated) interaction between GLD-1 and CGH-1, we tested if the two proteins may be functionally related. Because we were unable to create a strain containing both *gld-1(q485)* and *cgh-1(ok492)* null mutations, the temperature-sensitive *cgh-1(tn691)* allele, hereafter called *cgh-1^ts^*, was used in many experiments. This is an antimorphic allele (Ikuko Yamamoto and David Greenstein, personal communication; Figure S3E), which nevertheless induces oocyte defects and sheet-like CAR-1 containing structures also observed in *cgh-1* null or *cgh-1(RNAi)* gonads (data not shown; [5], [39]). We initially confirmed that the loss of CGH-1 activity had no obvious effect on the levels and distribution of GLD-1 ([39] and Figures S3C and S4B), nor did the loss of GLD-1 affect CGH-1 ([15] and Figure S3D). By microarrays, we examined the abundance of mRNAs in gonads dissected from *cgh-1^ts^* mutants grown at the restrictive temperature, and compared the changes in mRNA levels between *gld-1* and *cgh-1^ts^* gonads (Table S1 and Figure 4; encircled transcripts were confirmed by RT-qPCR in subsequent figures). Importantly, we observed that similar transcripts were reduced in each mutant (Figure 4; Pearson correlation coefficient r = 0.426) and that 47% of the transcripts reduced in both *gld-1* and *cgh-1^ts^* mutants were also GLD-1 targets (Figure 4, GLD-1 targets are in red); about four-fold more than expected by chance (p<2.2e-39, t test; only 12% of all germline mRNAs are bound by GLD-1). To make sure that the observed changes in mRNA levels were not unique to the *cgh-1^ts^* allele, we additionally analyzed mRNA changes in animals subjected to *cgh-1* RNAi and in *cgh-1* null mutants, and obtained similar results (Table S1 and Figure S4A–S4D). Combined, these results suggest that the mRNAs stabilized by GLD-1 are also stabilized by CGH-1. For the purpose of this study, we refer to those transcripts simply as ‘co-regulated mRNAs’ (Table S2; mRNAs that are GLD-1 targets, and which are less abundant in both *gld-1* and *cgh-1^ts^* mutants).

### GLD-1 binding directly elicits translational repression and mRNA stabilization

**Figure 5:**
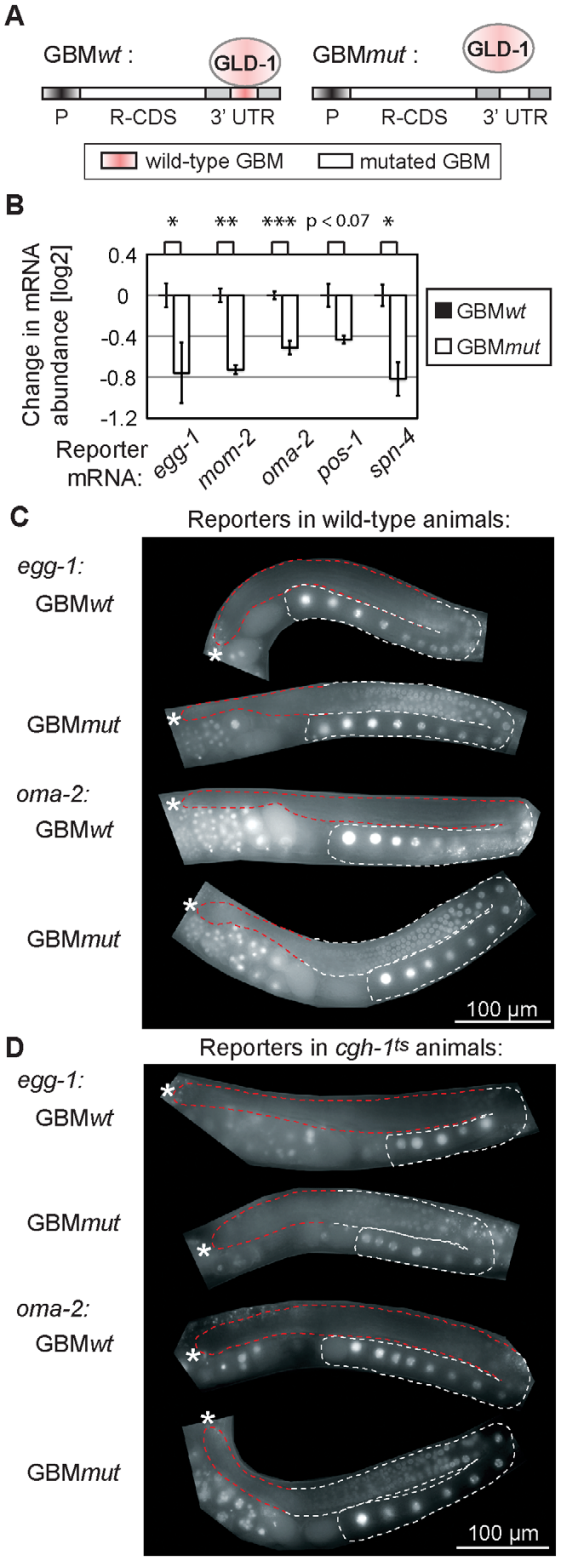
GLD-1 binding elicits mRNA stabilization and translational repression. (A) Schematic of reporters that were used to test the effect of GLD-1 binding motifs (GBMs) on mRNA stability and translation. P: a germ line-specific promoter (*mex-5*); R-CDS: reporter's coding sequence consisting of GFP fused to histone H2B; 3′UTR: GLD-1 target 3′ UTR containing either wild-type GBMs (GBM*wt*), or mutated GBMs (GBM*mut*) that no longer recruit GLD-1. (B) Mutating GBMs reduced mRNA levels of several reporters (shown is one reporter line per construct, for additional lines see S5A). Reporter mRNA levels were analyzed by RT-qPCR and normalized to *tbb-2* mRNA. Shown are mRNA level changes of GBM*mut* reporters relative to GBM*wt* reporters. One asterisk indicates p<0.05, two asterisks p<0.01, and three asterisks p<0.001 (p values were calculated with a t test). (C) Mutating GBMs caused *egg-1* and *oma-2* reporter de-repression in the medial gonad. Shown are photomicrographs of gonads (outlined; red highlighting repressed regions) from live, transgenic, and otherwise wild-type animals. (D) The same reporters were not (or additionally) de-repressed when crossed into *cgh-1^ts^* mutants. See also Figure S5B–S5C.

GLD-1 binds its mRNA targets via specific GLD-1 binding motifs (GBMs), which are mostly present in 3′ UTRs [31]. This enabled us to test a direct versus indirect role of GLD-1 in both mRNA repression and stabilization in wild-type animals, by creating a series of reporters containing either wild-type or mutated GBMs. Specifically, a constitutive germline promoter (*mex-5*) was used to drive transcription of GFP fused to histone H2B (which concentrates GFP in the nucleus to facilitate detection) [40]. The GFP reporter was fused to various 3′ UTRs of co-regulated mRNAs that either contained wild-type GBMs (GBM*wt*), allowing GLD-1 binding and regulation, or mutated GBMs (GBM*mut*), preventing GLD-1 binding and regulation (Figure 5A). We examined the effect of GLD-1 binding on mRNA stability by analyzing the levels of GBM*wt*/*mut* reporter pairs by RT-qPCR and found that, in each case, the GBM*mut* mRNA was less abundant than the corresponding GBM*wt* mRNA (Figure 5B and Figure S5A). Because these reporters were expressed and analyzed in wild-type animals, and mutated GBMs do not cause destabilization when introduced into the 3′ UTR of a non-target mRNA [31], these results suggest that GLD-1 stabilizes at least some of the co-regulated mRNAs by directly associating with their 3′ UTRs.

Expectedly, we observed that the GBM*mut* reporters were de-repressed in the medial germ line (Figure 5C and Figure S5B). To test the effect of CGH-1 on translational repression, we crossed the GBM*wt* and *mut* reporters into the *cgh-1^ts^* mutant, and additionally subjected reporter strains to *cgh-1* RNAi. Consistently with the observation that endogenous *glp-1* and *rme-2* mRNAs are not de-repressed in the medial gonad of *cgh-1(RNAi)* animals [39], we found that the GBM*wt* reporters were de-repressed in neither *cgh-1^ts^* nor *cgh-1(RNAi)* animals, nor were the GBM*mut* variants additionally de-repressed (Figure 5D and Figure S5C). These results suggest that, while CGH-1 contributes to the stabilization of GLD-1 targets, it does not appear to have a general role in their repression.

### GLD-1 and CGH-1 stabilize mRNAs independently of each other

**Figure 6:**
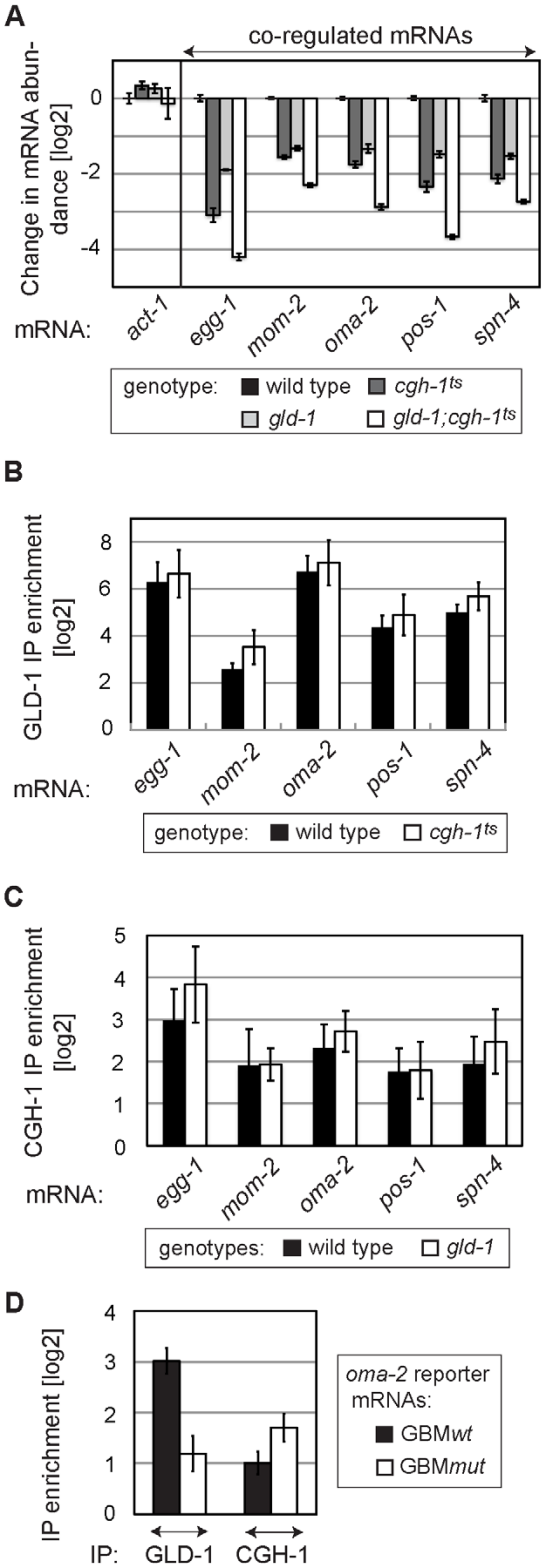
GLD-1 and CGH-1 stabilize mRNAs independently of each other. (A) GLD-1 and CGH-1 affect mRNA stability in an additive fashion. The levels of indicated mRNAs in wild-type, *gld-1*, *cgh-1^ts^*, and *gld-1;cgh-1^ts^* animals were measured by RT-qPCR and normalized to *tbb-2*. Shown are changes in mRNA abundance relative to the wild type. (B) GLD-1 binds co-regulated mRNAs in *cgh-1^ts^* animals. GLD-1 IPs were performed on lysates from wild-type and *cgh-1^ts^* animals and normalized to control (FLAG) IPs, to input mRNA levels, and finally to *tbb-2* mRNA. For similar IPs on *cgh-1* RNAi animals see Figure S6. (C) CGH-1 binds co-regulated mRNAs in the absence of GLD-1. CGH-1 IPs were performed on lysates from wild-type and *gld-1* animals and normalized to control (IgG) IPs, to input mRNA levels, and finally to *tbb-2* mRNA. (D) GLD-1 and CGH-1 are recruited to an mRNA independently of each other. GLD-1 and CGH-1 IPs were performed on lysates from *oma-2* GBM*wt* and GBM*mut* reporter-expressing animals and normalized to control IPs (FLAG and IgG respectively), to input mRNA levels and to *tbb-2* mRNA.

GLD-1 and CGH-1 may depend on each other for mRNA stabilization or function independently. To test whether GLD-1 and CGH-1 affect mRNA stability in an additive fashion, we determined the levels of endogenous co-regulated mRNAs in *gld-1* and *cgh-1^ts^* single, and *gld-1; cgh-1^ts^* double mutants. We found that mRNA levels, which were reduced in both single mutants, were even further reduced in the *gld-1; cgh-1^ts^* double mutant (Figure 6A), suggesting that GLD-1 and CGH-1 may stabilize mRNAs by acting in parallel pathways. Furthermore, to investigate if GLD-1 and CGH-1 depend on each other for mRNA binding, endogenous co-regulated mRNAs were co-precipitated with GLD-1 and CGH-1, from extracts of wild-type and mutant animals. One caveat of this analysis is that the RNA levels in mutant animals are reduced and obtained values need to be normalized to the corresponding input levels. By this approach, we observed that GLD-1 could still bind mRNAs in *cgh-1^ts^* and *cgh-1(RNAi)* animals, and likewise CGH-1 could bind mRNAs in the absence of GLD-1 (Figure 6B–6C and Figure S6). To test this further, we IP-ed GBM*wt* and GBM*mut* variants of the co-regulated *oma-2* reporter with GLD-1 and CGH-1 from worm lysates. As expected, we found that mutating GBMs in the *oma-2* 3′UTR dramatically decreased GLD-1 binding (Figure 6D). In contrast, we observed no reduction in CGH-1 binding (Figure 6D). Together, these results suggest that GLD-1 and CGH-1 are recruited to an mRNA independently of each other and that, although GLD-1 and CGH-1 stabilize largely the same mRNAs, their contributions appear to be distinct.

### GLD-1 and CGH-1 co-regulated mRNAs encode OET regulators and accumulate in oocytes

**Figure 7:**
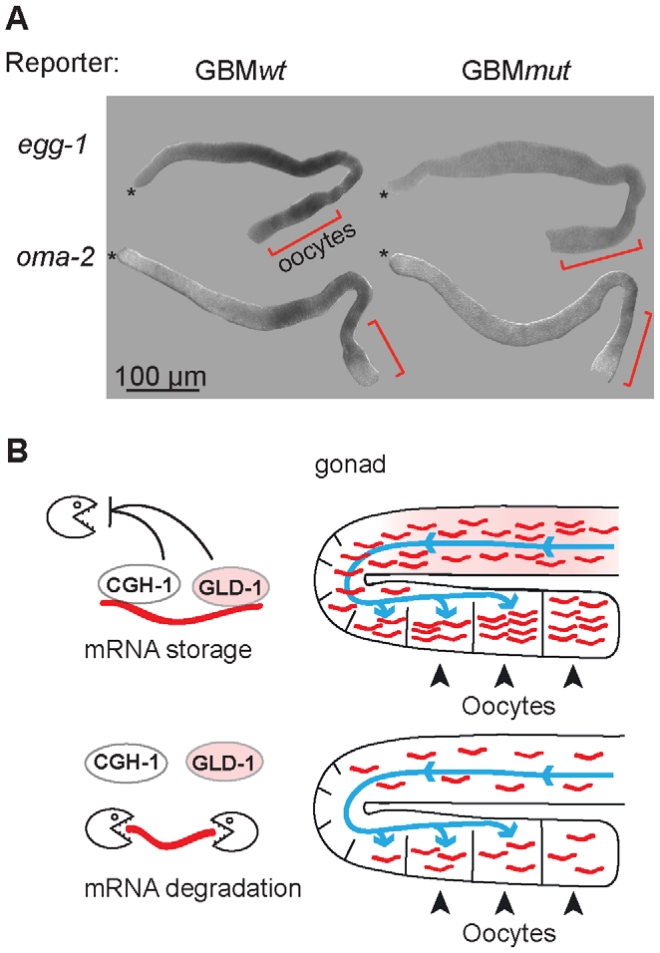
GLD-1 and CGH-1 co-regulated mRNAs accumulate in oocytes. (A) The expression patterns of GBM*wt* and GBM*mut* reporters as determined by in situ hybridization against *gfp* RNA. Wild-type gonads, not expressing a GFP reporter, were negative. Oocytes are indicated with brackets. (B) Model of how GLD-1 and CGH-1 mediated mRNA stabilization may lead to mRNA accumulation in oocytes. The blue lines indicate a general influx of the cytoplasm from undifferentiated germ cells to oocytes as reported by Wolke et al, 2007. GLD-1 and CGH-1 mediated protection in the medial part ensures that mRNAs are transported into oocytes (top). In the absence of this protection, mRNAs are degraded by an unknown mechanism(s) and thus fail to accumulate in oocytes.

Many of the co-regulated mRNAs encode proteins that have been studied in at least some detail. Remarkably, most of them (34/38) are important during the oocyte-to-embryo transition (Table S2). Some of these proteins function specifically during oogenesis (for example PUF-5; [35]), fertilization (EGG-1; [41]), or early embryogenesis (POS-1; [42]). Others, such as OMA-2, function at multiple times during OET [43]–[45]. Thus, GLD-1 and CGH-1 appear to stabilize messages related by their function in promoting OET. This was unexpected, because GLD-1 is expressed in the medial germ line but is absent from oocytes. To examine this seeming discrepancy, we tested by *in situ* hybridization whether the reporters of co-regulated mRNAs accumulate in oocytes in a GBM-dependent manner. Indeed, we found that the GBM*mut* reporters were less abundant not only in the medial, GLD-1 expressing part of the gonad, but also in the proximal gonad, suggesting that GLD-1 mediated stabilization is important for OET mRNA accumulation in oocytes (Figure 7A; position of oocytes is indicated by red brackets; see discussion).

## Discussion

### GLD-1 mediated translational repression

Previously, we identified hundreds of germline transcripts associated with GLD-1 [31]. Although the precise mechanism(s) remain unknown, here we present evidence that in general these messages are repressed by GLD-1, consistently with a recent report describing global protein changes in GLD-1 depleted animals [46]. We found that GLD-1 interacts with components of repressive germline mRNA complexes, including the DDX6 helicase CGH-1. In *Xenopus* and *Drosophila*, similar complexes also contain eIF4E-binding proteins (4E-BPs), suggesting that they repress translation by interfering with the assembly of the basic translation initiation factor eIF4F [12], [14], [47]–[50]. Interestingly, in *Drosophila*, the same proteins have also been implicated in *oskar* mRNA repression by a cap-independent mechanism, presumably by sequestering mRNAs away from the translation machinery [51]. Because we found neither basic initiation factors nor 4E-BPs among GLD-1 interacting proteins, one possibility is that GLD-1 represses its targets via a similar ‘sequestering’ mechanism, which might also protect them from the decay machinery. However, since GLD-1 appears to stabilize only a subset of its targets, and CGH-1 seems to protect but not repress them, translational repression and mRNA stabilization of co-regulated mRNAs are not necessarily coupled.

### GLD-1 and CGH-1 mediated mRNA stabilization

Our findings suggest that, in addition to repressing translation, GLD-1 stabilizes a subpopulation of its targets. The most compelling evidence comes from the GBM+/− reporter studies, which directly demonstrate that GLD-1 binding can stabilize a target mRNA. However, we noticed that the changes in mRNA levels induced by GBM mutations were smaller than the changes in the endogenous mRNAs observed between wild type and *gld-1* mutants. This could be due to a number of differences between the synthetic and endogenous mRNAs (such as expression from different promoters, splicing, potential additional regulatory motifs, etc.) or reflect a stronger decrease in mRNA levels in the mutant due to indirect effects. Thus, the precise magnitude of GLD-1 mediated stabilization remains to be determined. Besides GLD-1, our findings additionally implicate CGH-1 in the stabilization of some GLD-1 targets, but suggest that the two proteins regulate mRNA stability independently of each other. Possible interpretations of this data are that these proteins largely associate with separate cytoplasmic pools of mRNAs and/or protect mRNAs in different parts of the gonad. Yet, inconsistently with the later scenario, we noticed that the levels of several mRNAs tested by in situ hybridization were reduced also in the medial, GLD-1 expressing parts of *cgh-1^ts^* gonads (our unpublished observation). Interestingly, while CGH-1 appears to affect the stability of specific GLD-1 targets, it may be dispensable for their repression. This contrasts with the function of DDX6 helicases in translational repression in some models [12], [48], [49] but agrees with the role of the protist DDX6-like helicase, DOZI, which stabilizes repressed transcripts in female gametocytes [52]. Thus, either DDX6 helicases have distinct roles in different organisms, or their function in mRNA stabilization and/or mRNA repression depends on a specific mRNA.

### The flip side of OET mRNA stabilization—degradation of unprotected transcripts

Our finding, that GLD-1 and CGH-1-dependent stabilization may be important for efficient accumulation of OET transcripts, implies that the decay machinery responsible for the degradation of unprotected mRNAs is active in the germ line. Two GLD-1 targets containing upstream open reading frames are thought to be protected by GLD-1 from nonsense-mediated decay (NMD) [37]. However, we found no evidence that the degradation of unprotected GLD-1 targets described here depends on NMD (Figure S7). Interestingly, many OET mRNAs protected by GLD-1 and CGH-1 are degraded in early embryos (our unpublished observation). Thus, one possibility is that the machinery degrading maternal transcripts in the embryo degrades also unprotected OET mRNAs in the germ line. In *D. melanogaster*, the embryonic degradation of maternal mRNAs requires the protein Smaug and miRNAs [53]–[55]. Whether related factors degrade maternal mRNAs in the *C. elegans* embryo and/or unprotected OET mRNAs in the gonad remains to be tested.

### GLD-1– and CGH-1–dependent accumulation of OET mRNAs in oocytes

Intriguingly, our findings suggest that GLD-1 dependent stabilization of mRNAs is important for their accumulation in oocytes, i.e. in cells in which GLD-1 is no longer present. In *C. elegans*, oocyte growth depends on an influx of cytoplasmic material originating in undifferentiated, GLD-1 expressing cells, which may be analogous to the cytoplasmic transport from nurse cells into oocytes in the *Drosophila* ovary [56]. Thus, one explanation for GLD-1 dependent accumulation of mRNAs in oocytes is that GLD-1 binding protects mRNAs before and/or during their transport into growing oocytes (Figure 7B). Once in oocytes, these mRNAs might be stable due to a general suppression of mRNA decay, as described in *Xenopus* oocytes [2], [3]. Alternatively, GLD-1 might only be required for the initiation but not the maintenance of mRNA protection, which in oocytes may depend on CGH-1 and/or other RBPs.

## Materials and Methods

### Nematode culture, RNAi, mutants, transgenic strains, and worm sorting

Animals were typically maintained at 25°C using standard procedures, unless indicated otherwise. The temperature sensitive strain *cgh-1(tn691)* was maintained at 15°C and shifted to 25°C as L4 larvae for subsequent analysis of adult animals. Synchronous cultures were obtained by collecting eggs from bleached adults and synchronizing larvae by starvation before feeding. In all experiments young adults that produced oocytes but not yet embryos were analyzed. For RNAi experiments, we used the Open Biosystems *cgh-1* and *smg-2* bacterial strains and, as a control, bacteria harboring an ‘empty’ vector. Larvae were transferred to RNAi feeding plates directly after synchronization and animals were cultured at 25°C.

The following mutant and transgenic strains have been described previously: *cgh-1(ok492)/hT2[qIs48]*; *gld-1(q485)/hT2[qIs48]*; *rrrSi 38/39/40 [mex-5 pro::PEST:GFP-H2B::oma-2 3′UTR; unc-119(+)]II; and rrrSi 53/54/56[mex-5 pro::PEST:GFP-H2B::oma-2 GBMmut 3′UTR; unc-119(+)]II* [11], [31], [57]. The *cgh-1(tn691)* strain was obtained from CGC (DG1701); the cgh-1(tn691) mutation induces 100% sterility at the restrictive temperature (25°C).

To minimize variation between ‘GBM*wt*’ and ‘GBM*mut*’ pairs of reporters, transgenic strains were created by Mos1 transposase mediated Single Copy gene Insertion (MosSCI) into a single genomic locus as previously described [31], [58]. GBM mutations introduced are shown in Table S3. Oligos used to amplify 3′ UTR sequences (from the STOP codon to 50 bp downstream of the polyA site) are described in Table S4. All strains were outcrossed at least twice against wild-type animals before being analyzed. Table S5 shows all reporter strains utilized in this study. The 7 nt substitution that was used to mutate GBMs (in GBM*mut* reporters) does not by itself destabilize mRNA [31].

We used the COPAS Biosort from Union Biometrica to separate homozygous GFP (−) *gld-1* mutants from heterozygous GFP (+) *gld-1(q485)/hT2[qIs48]* animals.

### Polysome profile analysis and isolation of RNA and proteins

The assay was performed as previously described [59], with the following changes. Synchronized worms were harvested as young adults, frozen in 100 µl ‘worm pellet’ aliquots. Subsequently, each aliquot was re-suspended in 500 µl lysis buffer. An initial centrifugation step was included (5 min at 5000 g, 4°C) and worm lysates were layered on 5% (w/v) to 45% (w/v) sucrose gradients. To correct for variations in RNA isolation and reverse transcription efficiency between sucrose fractions, we added 2 µg of total RNA from mouse brain (Stratagene) to each fraction. RNA from fractions was extracted using TRIzol (Invitrogen) according to the manufacturer's recommendations. RNA integrity was confirmed on ethidium bromide-stained agarose gels before proceeding to RT. Proteins from fractions were isolated by chloroform/methanol precipitation and investigated by western blotting. To analyze mRNAs by tiling arrays, we extracted RNA from pooled fractions 8 to 12 (polysomal) and fractions 1–12 (total), in four biological replicates.

### RNA isolation from dissected gonads, whole animals, or RNAi–treated animals

50 gonads from wild-type, *gld-1(q485)*, and *cgh-1(tn691)* worms were dissected in triplicates in M9 buffer for tiling array analysis. The PicoPure RNA Isolation Kit was used according to the manufacturer's recommendations to extract RNA from gonads (Figure 2A and Figure 4). To analyze the mRNA abundance of various strains, RNA from 30 animals was extracted with the PicoPure RNA Isolation Kit (Figure 2B, Figure 5B, Figure 6A; Figures S4A, S5A, S7). To determine mRNA levels in mock and *cgh-1* RNAi treated animals, RNA was Trizol extracted from Input IP samples according to the manufacturer's recommendations.

### Antibodies

Peptides (Bachem) were used to generate mouse monoclonal antibodies according to standard procedures (PAB-1 = aa 542–560; GLD-1 = aa 65–79). PAB-1 antibody was diluted 1∶50 for western blot analysis. The mouse monoclonal GLD-1 antibody was used for immunoprecipitation (100 µl per reaction). Rabbit polyclonal GLD-1 antibody was used for western blot analysis (1∶50 dilution) and immunostaining (1∶500 dilution) [38]. Additional antibodies used: ACT-1 (MAB1501, Chemicon), CAR-1, CGH-1 [11], FLAG M2 (Sigma), GLH-1 [60], Myc (9E10), PGL-1 [61], GLH-1 [62].

### Immunoprecipitation and analysis of co-precipitated RNA

GLD-1 and CGH-1 immunoprecipitations were performed as previously described [5], [26], [31]. To globally identify GLD-1 targets by tiling arrays we compared anti-GLD-1 IPs with anti-Myc IPs. To determine GLD-1 mRNA binding in *cgh-1^ts^* and *cgh-1(RNAi)* animals, and CGH-1 mRNA binding in *gld-1* animals we compared anti-GLD-1 IPs with anti-FLAG IPs, and anti-CGH-1 IPs with anti-IgG IPs. RNA was eluted from beads with TRIzol. Precipitation efficiency was enhanced by adding 5 µg total RNA from mouse brain (Stratagene) to each IP sample.

### GLD-1 immunoprecipitation and analysis of co-precipitated proteins

GLD-1-associated proteins were identified by comparing anti-GLD-1 IPs with anti-FLAG IPs. RNase treated IP samples were incubated with 0.1 mg/ml RNase A (Qiagen) for 15 minutes at 37°C. Proteins were separated by SDS-PAGE and Coomassie stained. Bands were cut, washed and in-gel digested with trypsin overnight at 37°C. Tryptic peptides were separated on an Agilent 1100 nanoLC system (Agilent Technologies) coupled to an LTQ Orbitrap Velos hybrid mass spectrometer (Thermo Scientific). The LC system was equipped with a Peptide CapTrap column (Michrom BioResources, Inc.) and a capillary column with integrated nanospray tip (75 mm i.d.×100 mm, Swiss BioAnalytics AG) filled with MagicC18 (Michrom Bioresources, Inc.). Elution was performed with a gradient of 0–45% solvent B in 30 min at a flow rate of 400 nl/min. Solvent A consisted of 0.1% formic acid/2% acetonitrile, solvent B was composed of 0.1% formic acid/80% acetonitrile. The mass spectrometer operated in positive mode using the top 20 DDA method. Peptides were identified searching UniProt 15.14 using Mascot Distiller 2.3 and Mascot 2.2 (Matrix Science). Results were compiled in Scaffold 2.06. (Proteome Software).

### RT–qPCR

Reverse transcription reactions were performed using the ImProm-II Reverse Transcription System (Promega). To ensure that we are detecting full-length, polyadenylated transcripts we used oligo dT_(15)_ primers for RT reactions on RNA from polysome profile fractions. Identical results were obtained using random hexamer oligonucleotides. To compare total mRNA levels and analyze co-immunoprecipitated RNA, cDNA was generated using random hexamer primers. qPCR reactions were performed as described previously [26]. At least one primer in each pair is specific for an exon-exon junction (Table S4). Mouse RNA (*Cyt-c*) was added to polysomal fractions before RNA isolation and RT, allowing us to normalize all obtained qPCR results to *Cyt-c*, thereby correcting for variations in RNA isolation and RT. To compare mRNA levels between different mutants and analyze co-immunoprecipitated RNA, qPCR results were normalized as indicated.

### RNA hybridization to tiling arrays

300 ng of RNA (pooled gradient fractions, IP, RNAi treated animal extracts) or 5 µl of RNA (corresponding to 25 dissected gonads and isolated with the PicoPure Kit) were amplified once into dsDNA. 7.5 µg of cDNA was subsequently fragmented and labeled according to the “GeneChip Expression Analysis Technical Manual" (Affymetrix). 6 µg of fragmented and labeled DNA were hybridized to the Affymetrix *C. elegans* tiling array chip according to the Affymetrix Expression Analysis Technical Manual. Microarray sample preparation, hybridization and scanning were performed in the FMI genomics facility.

### Analysis of tiling array data

Tiling arrays were processed in R (www.r-project.org; [63]) using bioconductor [64], and the packages tilingArray [65] and preprocessCore. The arrays were RMA background corrected and log2 transformed on the oligo level using the command:
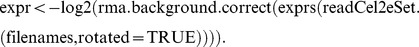
We mapped the oligos from the tiling array (bpmap file from www.affymetrix.com) to the *C. elegans* genome assembly ce6 (www.genome.ucsc.edu) using bowtie [66] allowing no error and unique mapping position. Expression of individual transcripts was calculated by intersecting the genomic positions of oligos with transcript annotation (WormBase WS190) and averaging the intensity of the respective oligos. Quantile normalization: each of the datasets was processed with an individual quantile normalization scheme. For IP experiments, no quantile normalization was performed as the distribution between GLD-1 IPs and control IPs differs substantially. In the case of the polysome dataset (containing polysome and total RNA samples) quantile normalization was performed twice. Once containing all the polysome samples and once for all the total RNA samples. The dataset containing either total RNA from purified gonads or total RNA from mock and *cgh-1(RNAi)* treated animals were each quantile normalized in one single step.

Averages, fold changes (including polysomal shifts) and standard deviations of all analyses are shown in Table S6. The p values in Figure 1D, Figure 4, Figure 5B; Figures S1E, S2 and S5A were calculated with a t test. To further investigate co-regulated mRNAs, we used the following cut-offs: *gld-1*/wild-type [log2]<−1; *cgh-1^ts^*/wild-type [log2]<−0.5. Data discussed in this publication has been deposited in NCBI's Gene Expression Omnibus and is accessible through GEO Series accession number GSE33084 (http://www.ncbi.nlm.nih.gov/geo/query/acc.cgi?acc=GSE33084).

### RNA in situ hybridization, immunolocalization, and microscopy

RNA in situ hybridization was performed and analyzed as previously described [26]. The probes generated from cDNA correspond to 1–714 (*gfp*) (Table S4). *gfp* antisense control on wild-type animals was included and negative. More than 60 gonads were scored and we obtained the following numbers (n = number of scored gonads):

*oma-2* GBM*wt* (n = 85; 27% strong; 65% medium; 6% weak); shown is a medium stained gonad

*oma-2* GBM*mut* (n = 65; 12% medium; 88% weak); shown is a weakly stained gonad

*egg-1* GBM*wt* (n = 70; 16% strong; 81% medium; 3% weak); shown is a medium stained gonad

*egg-1* GBM*mut* (n = 68; 35% medium; 65% weak); shown is a weakly stained gonad

Unless indicated otherwise, images were captured with a Zeiss AxioImager Z1 microscope, equipped with an Axiocam MRm REV2 CCD camera. Images were acquired in the linear mode of the Axiovision software (Zeiss) and processed with Adobe Photoshop CS4 in an identical manner.

### Confocal microscopy and deconvolution

An LSM700 confocal microscope equipped with a Plan-Apochromat 63×/1.40 Oil DIC M27 objective was used to capture images with a voxel size of 0.052 µm×0.052 µm×0.2 µm (x, y, z). Used lasers: track 1: 405 nm (2%) and 555 nm (10%); track 2: 488 nm (4%). Beam splitters: MBS 405/488/555/639; DBS1: 531 nm (track1) and 578 nm (track 2). Filters: SP490 (Track 1,Channel 1); LP 560 (Track 1, Channel 2); 0–587 (Track 2, Channel 1). Pinhole: 40 µm (Track 1); 41 µm (Track 2). Pictures were deconvolved with the Huygens software, using Remote Manager v1.2.3, a SNR of 8, 8, 8, 100 iterations, and the cmle deconvolution algorithm (quality change stopping criterion: 0.1). Deconvolved images were processed in Imaris XP 7.1.1 using the coloc function. In Figure S3C, only 5.41% of the total data set voxels co-localize.

## Supporting Information

Figure S1 Global survey of GLD-1 dependent mRNA repression. (A) A typical polysome profile derived from young (non-gravid) adults. Ultracentrifuged worm extracts were fractionated into 12 fractions. Arrows indicate the positions of monosomes (fraction 7) and polysomes (fractions 8–12). The bottom picture shows total RNA isolated from each fraction resolved on an agarose gel. (B) Polysome profile of EDTA-treated extracts from young adults. Polysomes (fractions 8–12) and monosomes (fraction 7) are disrupted by EDTA treatment. The integrity of total RNA from individual fractions was confirmed on an agarose gel. (C) The polysomal association of mRNAs is EDTA-sensitive. RNA levels in each fraction were measured by reverse-transcription and quantitative PCR (RT-qPCR), and normalized to mouse RNA that was added to each fraction. Shown are polysomal associations (fractions 8–12), normalized to total RNA (fractions 1–12). The polysomal association of mRNAs decreased upon EDTA treatment, suggesting that mRNAs present in the heavy fractions are associated with polysomes and actively translated. (D) Identification of GLD-1 associated mRNAs. 930 mRNAs (above the red line) were >3 fold enriched in GLD-1 IPs, compared to control MYC IPs. Co-IPed mRNAs were analyzed by tiling arrays. Each dot in this and subsequent plots represents a single transcript. Similar mRNAs co-purified with a FLAG and GFP-tagged GLD-1 (Pearson correlation coefficient r = 0.838), as previously described [31]. (E) GLD-1 but not FBF-1 targets shift to polysomes in *gld-1* mutants. The data sets of this study and [34] were merged and subsequently analyzed (because different arrays were utilized, the merge caused a reduction of both data sets). Box plots represent the distribution of polysomal/total mRNA ratios for GLD-1 and FBF targets (mRNAs that were >3 fold enriched in FBF IPs, compared to control IPs [34]) and non-targets in wild-type and *gld-1* animals. Only GLD-1 targets shift significantly to polysomes in *gld-1* animals. Sample sizes (n) and p values are indicated. The p values were calculated with a t test.
(TIF)

Figure S2 GLD-1 dependent repression can be uncoupled from mRNA stabilization. Box plots represent the distribution of polysomal/total mRNA ratios in wild-type and *gld-1* mutant animals. GLD-1 targets were grouped into stabilized (*gld-1*/wt<log2(−1)) and non stabilized target mRNAs. Both groups of GLD-1 targets shift to polysomes in *gld-1* mutants. Sample sizes (n) and p values (t test) are indicated.
(TIF)

Figure S3 GLD-1 interacts with conserved components of RNA granules. (A) Proteins in GLD-1 and FLAG IPs were analyzed by mass spectrometry. Shown is the total number of assigned spectra of all peptides per protein. GLD-1 itself and a set of conserved RNA binding proteins were enriched in GLD-1 but not negative control (FLAG) IPs. Proteins such as VIT-2, VIT-6 and HSP-60 were equally enriched in both IPs. (B) GLD-1 and CGH-1 are largely present in distinct cytoplasmic foci. Confocal microscopy on dissected wild-type gonads that were immunostained for GLD-1 and CGH-1. Pictures were deconvolved and shown is a fragment from the medial germ line, whose approximate location is marked by the red square on the schematic gonad. Shown are pictures of CGH-1 and GLD-1distribution, and the merge of both without and with co-localized voxels (in yellow). (C) Loss of CGH-1 function does not affect GLD-1 protein levels. Total worm extracts from wild type and temperature-sensitive *cgh-1(tn691)* mutants were analyzed by western blotting. The temperature sensitive point mutant allele *tn691* only decreases CGH-1 levels at the restrictive temperature. (D) Loss of GLD-1 does not affect CGH-1 protein levels. Total worm extracts from wild-type and *gld-1(q485)* mutants were analyzed by western blotting. GLD-1 is not detectable, while CGH-1 and ACT-1 levels are not affected. (E) Analysis of the *cgh-1^ts^* allele. Animals of the indicated genotypes were shifted to 25°C at the L4 stage. Gonad defects were scored at the young adult stage.
(TIF)

Figure S4 CGH-1 is required for the accumulation of some GLD-1 targets. (A) The levels of co-regulated mRNAs decrease in different *cgh-1* mutants. The levels of indicated mRNAs in wild type, *cgh-1^ts^*, and *cgh-1(ok492)* null mutants were measured by RT-qPCR and normalized to *tbb-2*. Shown are changes in mRNA abundance relative to the wild type. (B) Confirmation of RNAi mediated CGH-1 depletion. Total worm extracts from mock and *cgh-1* RNAi treated animals were analyzed by western blotting. CGH-1 levels are decreased, while GLD-1 and ACT-1 levels are not affected. (C) The levels of co-regulated mRNAs decrease in *cgh-1(RNAi)* animals. The levels of indicated mRNAs in mock and *cgh-1* RNAi treated animals were measured by RT-qPCR and normalized to *tbb-2*. Shown are changes in mRNA abundance relative to mock treated animals. (D) Similar mRNAs are reduced in *cgh-1(RNAi)* animals and in *cgh-1^ts^* mutant gonads. The change in mRNA abundance in *cgh-1(RNAi)* animals was plotted against the change in mRNA abundance in *cgh-1^ts^* mutants (Pearson correlation coefficient r = 0.39). ‘co-regulated’ mRNAs are colored in red.
(TIF)

Figure S5 CGH-1 does not generally repress translation of GLD-1 targets. (A) GBM mutations in additional strains decrease reporter mRNA levels. Reporter mRNA levels were analyzed by RT-qPCR and normalized to *tbb-2* mRNA. Shown are changes in the mRNA abundance of GBM*mut* reporters relative to GBM*wt* reporters. One asterisk indicates p<0.05 and two asterisks p<0.01 (t test). (B) Mutating GBMs caused reporter de-repression in the medial gonad. Shown are photomicrographs of gonads (outlined; red highlighting repressed regions) from live, transgenic, mock RNAi treated animals. (C) The same reporters were not (or additionally) de-repressed in *cgh-1(RNAi)* animals. The bars correspond to 100 µm.
(TIF)

Figure S6 GLD-1 binds co-regulated mRNAs in the absence of CGH-1. GLD-1 IPs were performed on lysates from mock and *cgh-1* RNAi treated animals and normalized to control (FLAG) IPs, to input mRNA levels, and finally to *tbb-2* mRNA.
(TIF)

Figure S7 GLD-1 does not generally protect its targets from NMD. (A) Wild-type and *gld-1* mutant animals were treated with mock and *smg-2* RNAi. Shown are published NMD targets [37] and several co-regulated RNAs. While inactivating the NMD machinery in *gld-1* mutants prevents mRNA degradation of NMD targets, co-regulated mRNAs continue to be degraded in *gld-1* mutants.
(TIF)

Table S1 List of mRNAs analyzed by tiling arrays. Excel spread sheet showing the Wormbase Gene ID; [log2] values of the mRNA abundance in wild-type, *gld-1* and *cgh-1^ts^* gonad samples; [log2] values of the mRNA abundance in control and GLD-1 IP samples; [log2] values of total and polysomal mRNA abundance in wild-type and *gld-1* mutant animals; and [log2] values of the mRNA abundance in mock and *cgh-1* RNAi treated animals.
(XLS)

Table S2 List of ‘co-regulated’ mRNAs. Excel spread sheet showing the Wormbase Gene ID; the time point when these genes are first required according to wormbase; the gene public name; and [log2] values of GLD-1 IP enrichment, the change in mRNA abundance in *gld-1* mutants, the change in mRNA abundance in *cgh-1^ts^* mutants and the change in mRNA abundance in *cgh-1(RNAi)* animals.
(XLS)

Table S3 List of mutated GBMs in this study. Indicated are sequences before and after mutagenesis, the 3′-UTR that contains the GBM and the position of the GBM.
(XLS)

Table S4 List of oligos used in this study. Shown are names, function and oligo sequences.
(XLS)

Table S5 List of transgenic strains used in this study.
(XLS)

Table S6 Statistical analysis of Figure 1D and Figures S1E and S2. Shown are fold change [log2], average [log2] and standard deviation [log2].
(XLS)
