# Supplementary figures and images for: Genome-Wide Analysis of GLD-1–Mediated mRNA Regulation Suggests a Role in mRNA Storage
Source: PLoS Genet. 2012 May 31;8(5):e1002742. doi: 10.1371/journal.pgen.1002742 (PMC3364957; doi:10.1371/journal.pgen.1002742)

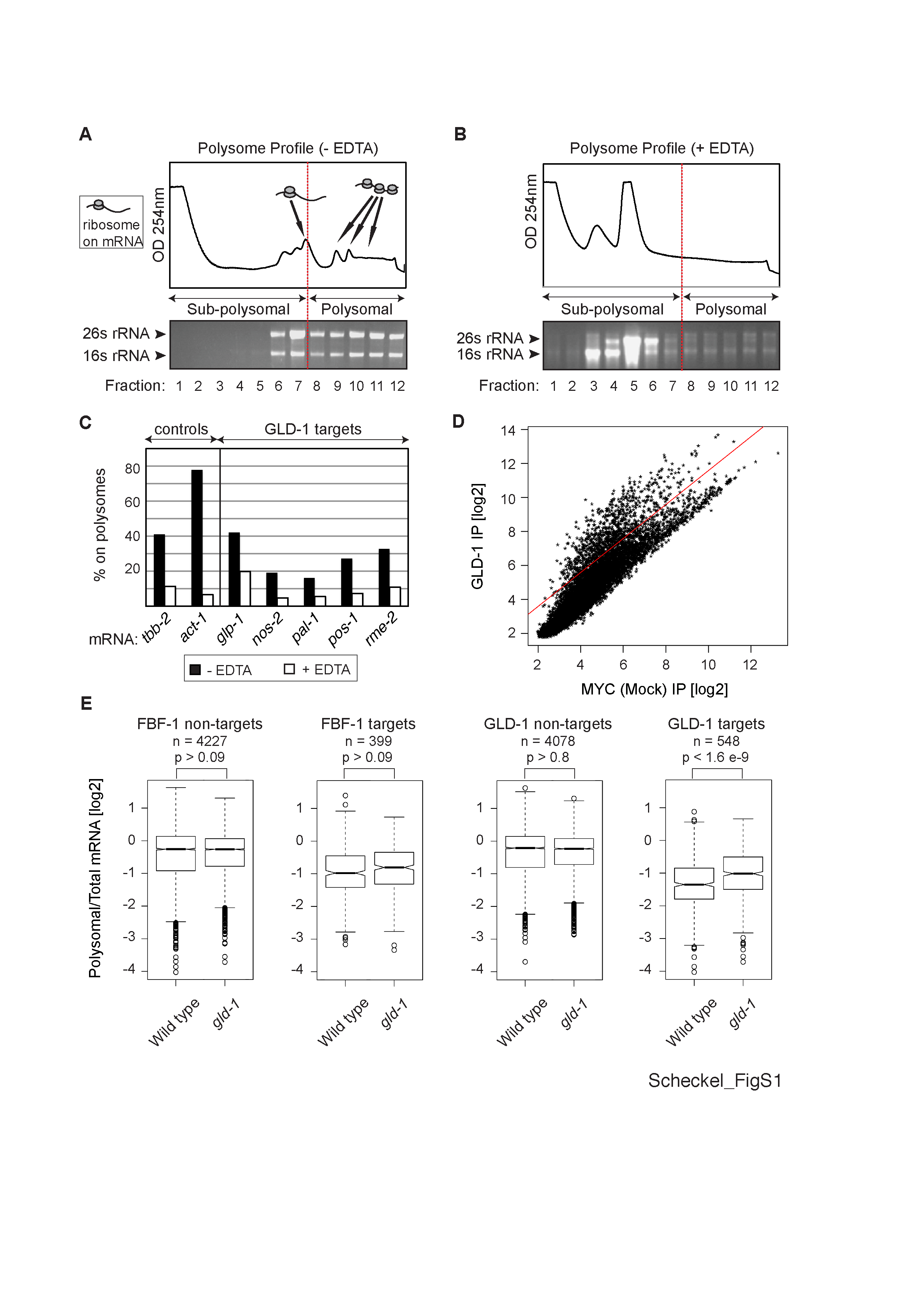

Supplement: Figure S1 — Global survey of GLD-1 dependent mRNA repression. (A) A typical polysome profile derived from young (non-gravid) adults. Ultracentrifuged worm extracts were fractionated into 12 fractions. Arrows indicate the positions of monosomes (fraction 7) and polysomes (fractions 8–12). The bottom picture shows total RNA isolated from each fraction resolved on an agarose gel. (B) Polysome profile of EDTA-treated extracts from young adults. Polysomes (fractions 8–12) and monosomes (fraction 7) are disrupted by EDTA treatment. The integrity of total RNA from individual fractions was confirmed on an agarose gel. (C) The polysomal association of mRNAs is EDTA-sensitive. RNA levels in each fraction were measured by reverse-transcription and quantitative PCR (RT-qPCR), and normalized to mouse RNA that was added to each fraction. Shown are polysomal associations (fractions 8–12), normalized to total RNA (fractions 1–12). The polysomal association of mRNAs decreased upon EDTA treatment, suggesting that mRNAs present in the heavy fractions are associated with polysomes and actively translated. (D) Identification of GLD-1 associated mRNAs. 930 mRNAs (above the red line) were >3 fold enriched in GLD-1 IPs, compared to control MYC IPs. Co-IPed mRNAs were analyzed by tiling arrays. Each dot in this and subsequent plots represents a single transcript. Similar mRNAs co-purified with a FLAG and GFP-tagged GLD-1 (Pearson correlation coefficient r = 0.838), as previously described [31]. (E) GLD-1 but not FBF-1 targets shift to polysomes in gld-1 mutants. The data sets of this study and [34] were merged and subsequently analyzed (because different arrays were utilized, the merge caused a reduction of both data sets). Box plots represent the distribution of polysomal/total mRNA ratios for GLD-1 and FBF targets (mRNAs that were >3 fold enriched in FBF IPs, compared to control IPs [34]) and non-targets in wild-type and gld-1 animals. Only GLD-1 targets shift significantly to polysomes in gld-1 anim [file pgen.1002742.s001.tif]

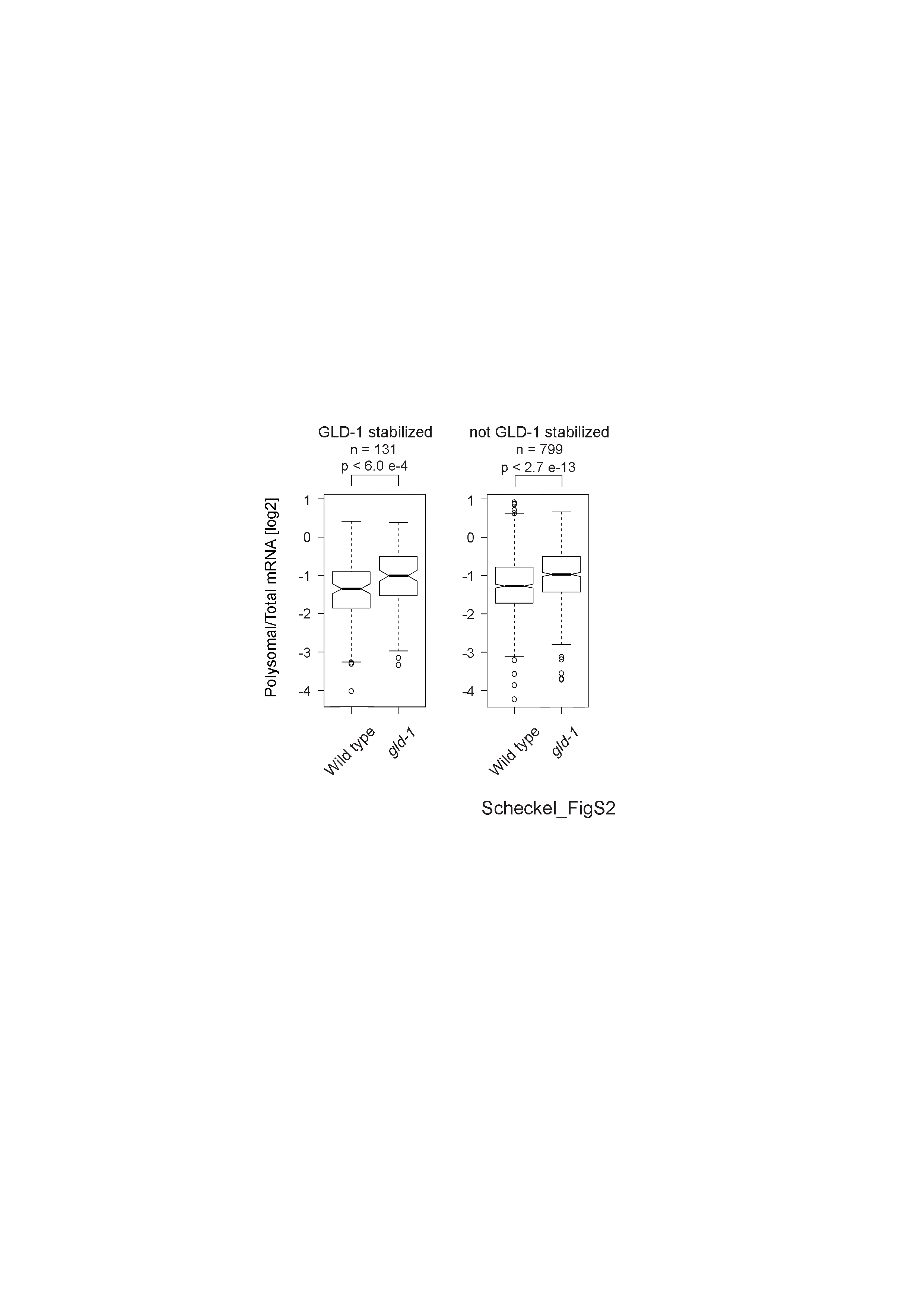

Supplement: Figure S2 — GLD-1 dependent repression can be uncoupled from mRNA stabilization. Box plots represent the distribution of polysomal/total mRNA ratios in wild-type and gld-1 mutant animals. GLD-1 targets were grouped into stabilized (gld-1/wt<log2(−1)) and non stabilized target mRNAs. Both groups of GLD-1 targets shift to polysomes in gld-1 mutants. Sample sizes (n) and p values (t test) are indicated. (TIF) [file pgen.1002742.s002.tif]

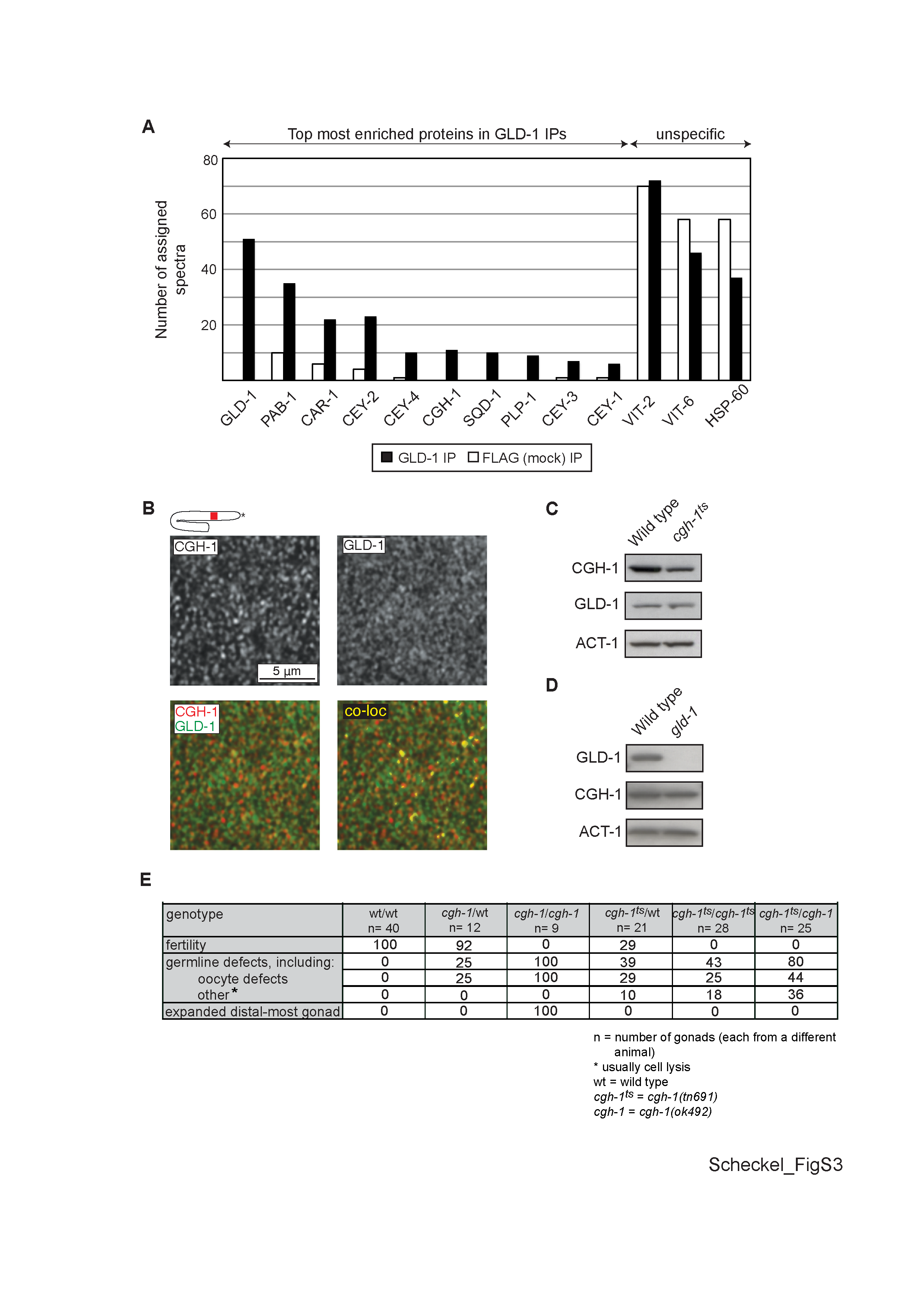

Supplement: Figure S3 — GLD-1 interacts with conserved components of RNA granules. (A) Proteins in GLD-1 and FLAG IPs were analyzed by mass spectrometry. Shown is the total number of assigned spectra of all peptides per protein. GLD-1 itself and a set of conserved RNA binding proteins were enriched in GLD-1 but not negative control (FLAG) IPs. Proteins such as VIT-2, VIT-6 and HSP-60 were equally enriched in both IPs. (B) GLD-1 and CGH-1 are largely present in distinct cytoplasmic foci. Confocal microscopy on dissected wild-type gonads that were immunostained for GLD-1 and CGH-1. Pictures were deconvolved and shown is a fragment from the medial germ line, whose approximate location is marked by the red square on the schematic gonad. Shown are pictures of CGH-1 and GLD-1distribution, and the merge of both without and with co-localized voxels (in yellow). (C) Loss of CGH-1 function does not affect GLD-1 protein levels. Total worm extracts from wild type and temperature-sensitive cgh-1(tn691) mutants were analyzed by western blotting. The temperature sensitive point mutant allele tn691 only decreases CGH-1 levels at the restrictive temperature. (D) Loss of GLD-1 does not affect CGH-1 protein levels. Total worm extracts from wild-type and gld-1(q485) mutants were analyzed by western blotting. GLD-1 is not detectable, while CGH-1 and ACT-1 levels are not affected. (E) Analysis of the cgh-1ts allele. Animals of the indicated genotypes were shifted to 25°C at the L4 stage. Gonad defects were scored at the young adult stage. (TIF) [file pgen.1002742.s003.tif]

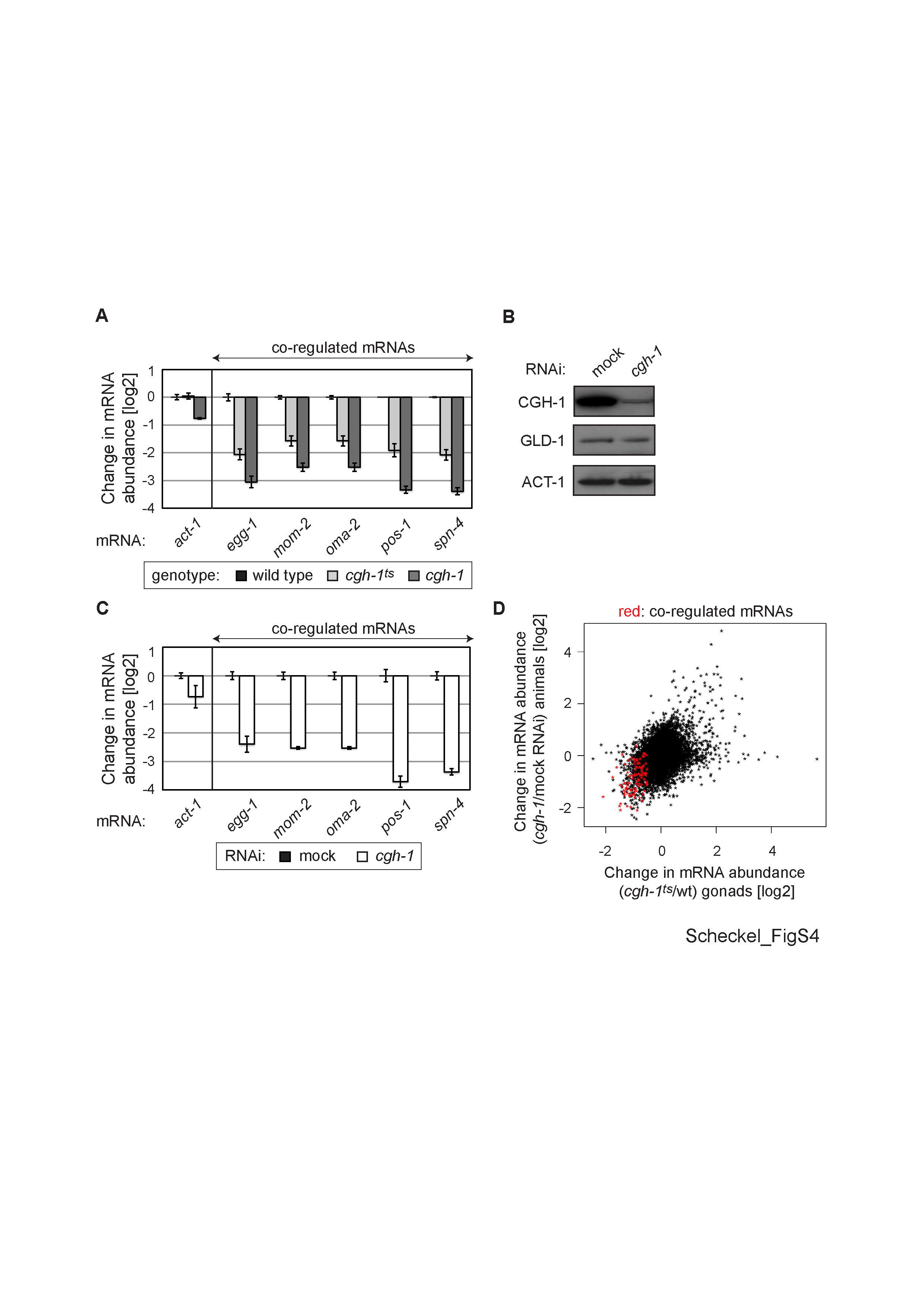

Supplement: Figure S4 — CGH-1 is required for the accumulation of some GLD-1 targets. (A) The levels of co-regulated mRNAs decrease in different cgh-1 mutants. The levels of indicated mRNAs in wild type, cgh-1ts, and cgh-1(ok492) null mutants were measured by RT-qPCR and normalized to tbb-2. Shown are changes in mRNA abundance relative to the wild type. (B) Confirmation of RNAi mediated CGH-1 depletion. Total worm extracts from mock and cgh-1 RNAi treated animals were analyzed by western blotting. CGH-1 levels are decreased, while GLD-1 and ACT-1 levels are not affected. (C) The levels of co-regulated mRNAs decrease in cgh-1(RNAi) animals. The levels of indicated mRNAs in mock and cgh-1 RNAi treated animals were measured by RT-qPCR and normalized to tbb-2. Shown are changes in mRNA abundance relative to mock treated animals. (D) Similar mRNAs are reduced in cgh-1(RNAi) animals and in cgh-1ts mutant gonads. The change in mRNA abundance in cgh-1(RNAi) animals was plotted against the change in mRNA abundance in cgh-1ts mutants (Pearson correlation coefficient r = 0.39). ‘co-regulated’ mRNAs are colored in red. (TIF) [file pgen.1002742.s004.tif]

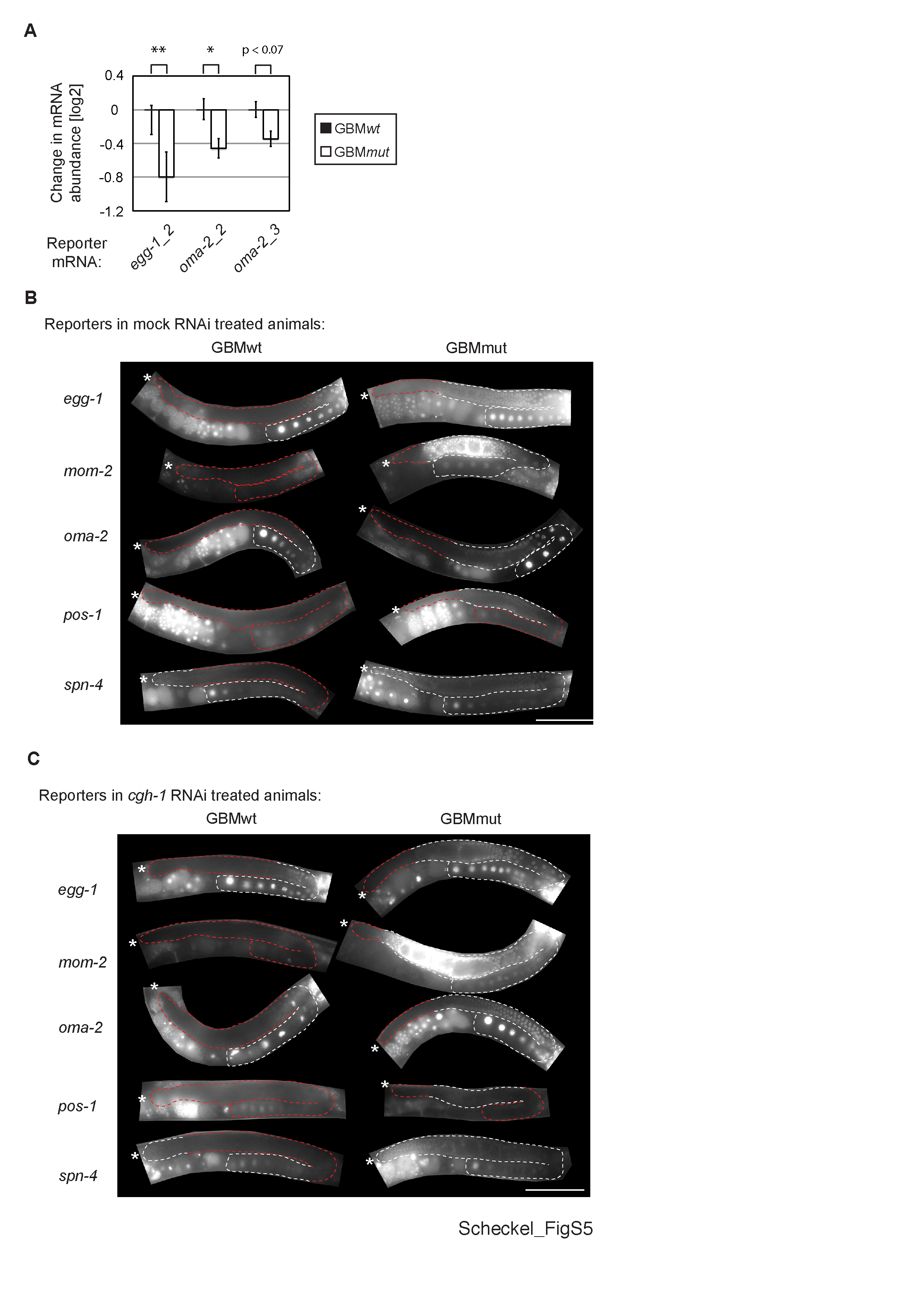

Supplement: Figure S5 — CGH-1 does not generally repress translation of GLD-1 targets. (A) GBM mutations in additional strains decrease reporter mRNA levels. Reporter mRNA levels were analyzed by RT-qPCR and normalized to tbb-2 mRNA. Shown are changes in the mRNA abundance of GBMmut reporters relative to GBMwt reporters. One asterisk indicates p<0.05 and two asterisks p<0.01 (t test). (B) Mutating GBMs caused reporter de-repression in the medial gonad. Shown are photomicrographs of gonads (outlined; red highlighting repressed regions) from live, transgenic, mock RNAi treated animals. (C) The same reporters were not (or additionally) de-repressed in cgh-1(RNAi) animals. The bars correspond to 100 µm. (TIF) [file pgen.1002742.s005.tif]

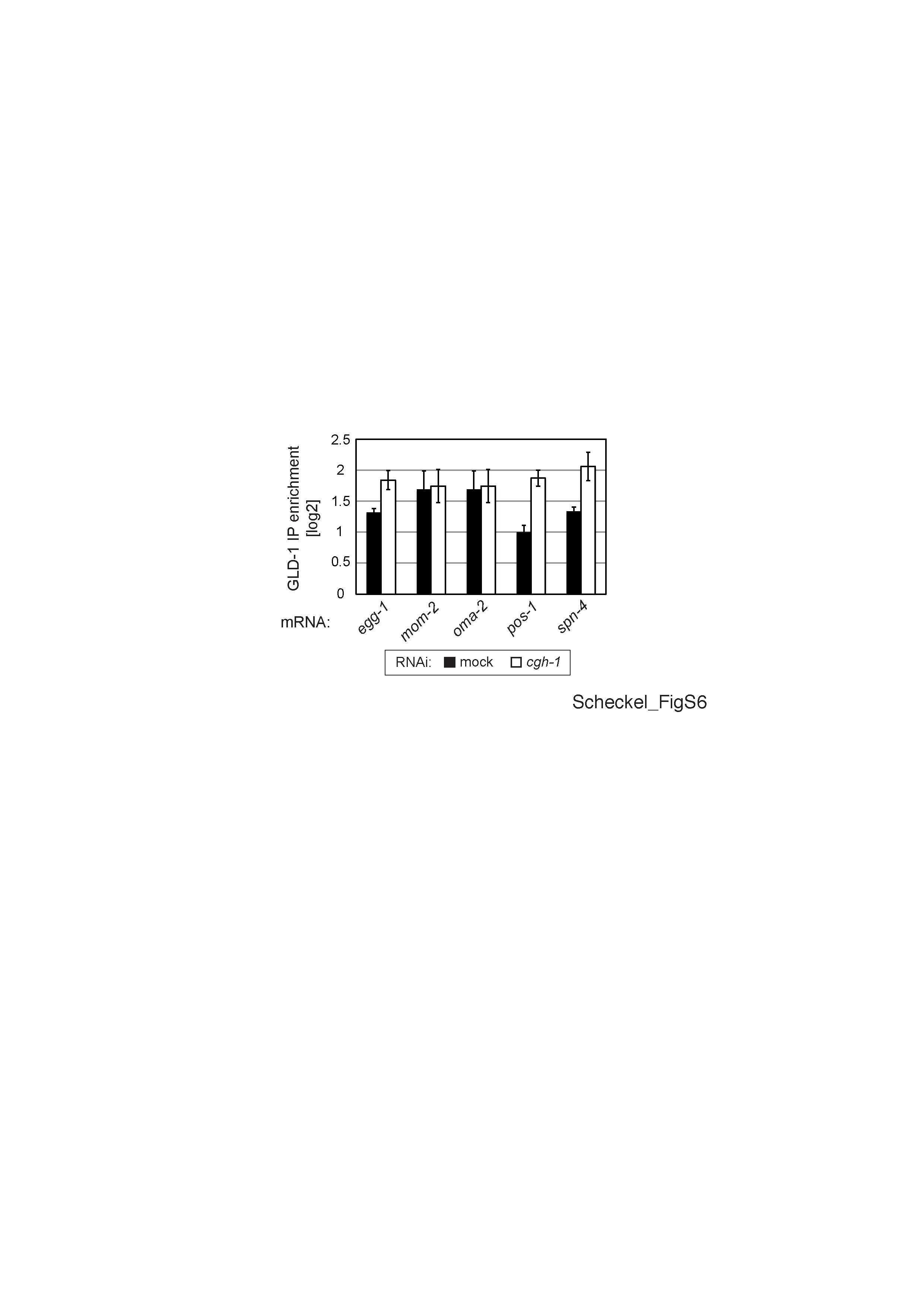

Supplement: Figure S6 — GLD-1 binds co-regulated mRNAs in the absence of CGH-1. GLD-1 IPs were performed on lysates from mock and cgh-1 RNAi treated animals and normalized to control (FLAG) IPs, to input mRNA levels, and finally to tbb-2 mRNA. (TIF) [file pgen.1002742.s006.tif]

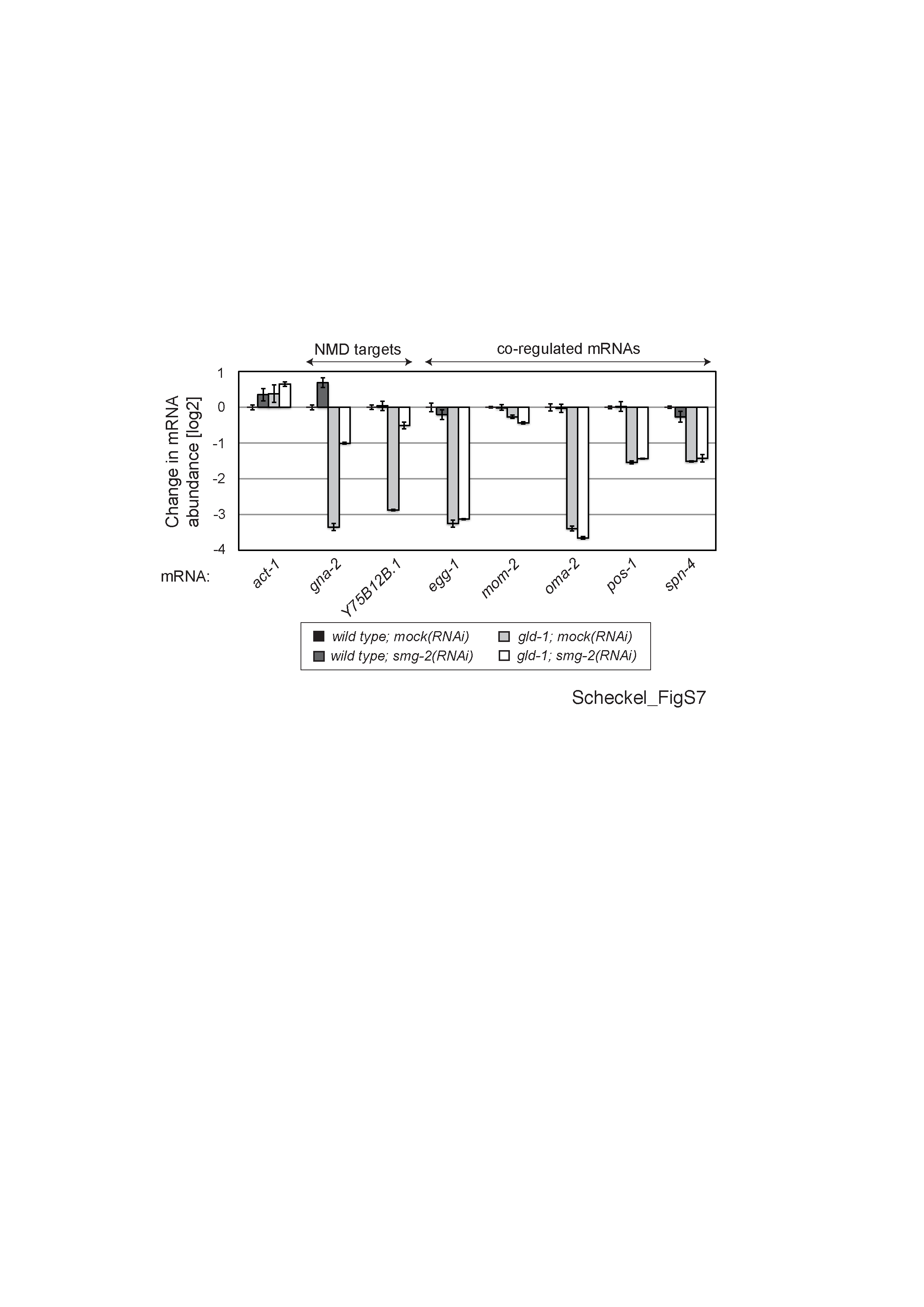

Supplement: Figure S7 — GLD-1 does not generally protect its targets from NMD. (A) Wild-type and gld-1 mutant animals were treated with mock and smg-2 RNAi. Shown are published NMD targets [37] and several co-regulated RNAs. While inactivating the NMD machinery in gld-1 mutants prevents mRNA degradation of NMD targets, co-regulated mRNAs continue to be degraded in gld-1 mutants. (TIF) [file pgen.1002742.s007.tif]
